# Empagliflozin and Rapid Kidney Function Decline Incidence in Type 2 Diabetes: An Exploratory Analysis From the EMPA-REG OUTCOME Trial

**DOI:** 10.1016/j.xkme.2023.100783

**Published:** 2023-12-18

**Authors:** Samy Hadjadj, Mark E. Cooper, Dominik Steubl, Michaela Petrini, Stefan Hantel, Michaela Mattheus, Christoph Wanner, Merlin C. Thomas

**Affiliations:** 1 Institut du thorax, INSERM, CNRS, Université Nantes, CHU Nantes, Nantes, France; 2 Department of Diabetes, Monash University, Melbourne, Australia; 3 Boehringer Ingelheim International GmbH, Ingelheim, Germany, and Department of Nephrology, Klinikum rechts der Isar, Technische Universität München, Munich, Germany; 4 Boehringer Ingelheim Pharmaceuticals, Inc, Ridgefield, Connecticut; 5 Boehringer Ingelheim Pharma GmbH & Co. KG, Biberach, Germany; 6 Boehringer Ingelheim Pharma GmbH & Co. KG, Ingelheim, Germany; 7 Department of Internal Medicine I, University Hospital Würzburg, Würzburg, Germany; 8 Department of Diabetes, Monash University, Melbourne, Australia

**Keywords:** Chronic kidney disease, diabetes mellitus, randomized controlled trials, estimated glomerular filtration rate, kidney function

## Abstract

**Rationale & Objective:**

Kidney function progressively declines in most patients with type 2 diabetes (T2DM). Many develop progressive chronic kidney disease (CKD), but some experience a more rapid decline, with a greater risk of kidney failure and cardiovascular disease. In EMPA-REG OUTCOME, empagliflozin was associated with slower kidney disease progression. This post hoc analysis evaluated the effect of empagliflozin (pooled doses) on the prevalence of a “rapid decliner” phenotype, defined by an annual estimated glomerular filtration rate (eGFR) decline of >3 mL/min/1.73 m^2^.

**Study Design:**

This was an exploratory analysis of EMPA-REG OUTCOME, a large randomized, double-blind, placebo-controlled trial in adults with T2DM, established cardiovascular disease and an eGFR of ≥30 mL/min/1.73 m^2^.

**Setting & Participants:**

Analysis was undertaken on 6,967 participants (99.2%) in whom serial eGFR data was available.

**Interventions:**

Patients were randomized (1:1:1) to empagliflozin 10 mg, 25 mg, or placebo in addition to standard of care.

**Outcomes:**

Annual change in eGFR over the maintenance phase of treatment (week 4 to last value on treatment) was calculated using linear regression models. Logistic regression analysis was used to investigate differences in rapid decline between the treatment groups.

**Results:**

Over the study period, a rapid decliner phenotype was observed in 188 (9.5%) participants receiving placebo and 134 (3.4%) receiving empagliflozin. After adjusting for other risk factors, this equated to a two-third reduction in odds (OR, 0.32; 95% CI, 0.25-0.40; *P* < 0.001) among participants receiving empagliflozin versus placebo. A comparable risk reduction was observed using a threshold of eGFR decline of >5 mL/min/1.73 m^2^/y (empagliflozin vs placebo, 43 [1.1%] vs 44 [2.2%] participants; OR, 0.47; 95% CI, 0.31-0.72; *P* < 0.001).

**Limitations:**

This is a post hoc analysis of a trial undertaken in participants with T2DM and CVD. Generalization of findings to other settings remains to be established.

**Conclusions:**

Patients receiving empagliflozin were significantly less likely to experience a rapid decline in eGFR over a median of 2.6 years of exposure to the study drug.

**Funding:**

The Boehringer Ingelheim and Eli Lilly and Company Diabetes Alliance.

**Trial Registration:**

clinicaltrials.gov ID: NCT01131676

## Plain-Language Summary

In most people with type 2 diabetes, their kidney function starts to decline over time. However, in some people, this can happen more rapidly, which can increase their risk of kidney or cardiovascular disease. A major study, EMPA-REG OUTCOME, has shown that empagliflozin, which helps to control blood sugar in people with type 2 diabetes, also reduced the risk of cardiovascular disease events and slowed the progression of kidney disease, when compared with people in the study who received placebo. In this new research from the same major study empagliflozin, compared with a placebo, was shown to reduce the risk of people having a rapid decline in their kidney function over the 3 years of the study.

Type 2 diabetes (T2DM) is associated with an accelerated decline in kidney function and the development of chronic kidney disease (CKD), owing to diabetes itself and/or its comorbid conditions, such as hypertension, dyslipidemia, obesity, acute kidney injury (AKI), atherosclerosis, and kidney ischemia,^1^ leading to an increased risk of kidney failure, major acute cardiovascular events, heart failure, and premature mortality.^2^ The effect of CKD on patient health, quality of life, cost of care, and ultimate prognosis in T2DM is profound. Indeed, excess mortality associated with T2DM appears to be largely confined to those with CKD, in which the presence of a reduced estimated glomerular filtration rate (eGFR), elevated urinary albumin excretion, and especially both, identifies patients who are at increased risk for adverse health outcomes.^3^ By contrast, those patients without signs of CKD appear to have a prognosis that is not very different from individuals without diabetes.^3^ Consequently, finding additional methods to prevent or slow the decline in kidney function is a key priority for the management of T2DM.^4^^,^^5^

Clinical trials have documented a lower risk of kidney outcomes and progression of kidney disease in participants using sodium/glucose cotransporter 2 (SGLT2) inhibitors when compared with placebo, although these initial studies were primarily designed as cardiovascular outcomes trials, and the kidney outcomes were only secondary or exploratory endpoints.^6^, ^7^, ^8^, ^9^ These findings have been confirmed in dedicated trials of patients with CKD both with or without T2DM.^10^, ^11^, ^12^ In addition, the rate of change of eGFR (ie, its decline) appears to be slower in participants receiving SGLT2 inhibitors compared with those without SGLT2 inhibitors on top of standard glucose lowering and cardiovascular (CV) therapies.^6^^,^^7^^,^^13^ For example, in the EMPA-REG OUTCOME trial, after an initial dip in the eGFR with treatment initiation, the mean eGFR remained stable in the empagliflozin-treated groups; however, declined steadily in the placebo group (*P* < 0.001 for both comparisons with placebo).^6^ The mechanisms underlying this observation remain to be established.

Small reductions in the eGFR slope of 0.5-1.0 mL/min/1.73 m^2^/y are generally associated with hazard ratios of ∼0.7 for clinical outcomes in cohorts and trials.^14^ Hence, the slower rate of eGFR decline seen in participants with versus without SGLT2 inhibitor treatment is likely to be clinically relevant, given the risks associated with a relatively rapid decline in the eGFR. We hypothesized that the apparent stability of kidney function in participants receiving empagliflozin may be associated with fewer participants experiencing a rapid decline in kidney function versus placebo over the course of the EMPA-REG OUTCOME trial. In an exploratory post hoc analysis considering the overall trial population, we aimed primarily to compare empagliflozin with placebo on rapid kidney function decline, defined by certain annual eGFR slope thresholds, and to analyze its determinants. Furthermore, we aimed to study how the annual changes translated to the individual time to projected kidney failure (time of first reaching eGFR value of ≤10 mL/min/1.73 m^2^, which is maintained), if a patient does not die, by applying a model to on-treatment data from week 4 onwards and extrapolating the eGFR course until 15 years after baseline. Another objective was to compare empagliflozin to a placebo on the incidence of AKI among patients with and without rapid eGFR decline to assess if rapid eGFR decline predisposes for AKI.

## Methods

### Trial Design and Oversight

The design and methods of the EMPA-REG OUTCOME trial have been described previously.^15^ Briefly, the study population included only patients who had T2DM, established cardiovascular disease, and an eGFR of ≥30 mL/min/1.73 m^2^ of body-surface area, according to the 4-variable Modification of Diet in Renal Disease (MDRD) Study equation. An independent ethics committee or institutional review board approved the clinical protocol at each participating center. All patients provided written informed consent before study entry. Patients were randomly assigned to receive either empagliflozin (at a dose of 10 mg or 25 mg) or placebo once daily in addition to standard care. For the purpose of the present analysis, results from both empagliflozin doses were pooled, unless otherwise indicated.

### Determination of the Rate of Change of eGFR and Rapid Decline

We used the MDRD Study equation to assess the eGFR at baseline and serially (at weeks 4, 12, 26, 52, and every 14 weeks thereafter) throughout the study and at 30 days after stopping treatment (ie, follow-up). Individual and average rate of change of eGFR per year was assessed by prespecified eGFR slope analyses using a random intercept and time coefficient model, as described previously by Wanner et al.^6^^,^^16^

Treatment with SGLT2 inhibitors including empagliflozin is associated with a reversible, largely hemodynamic initial decline in eGFR because of a reduction in the intraglomerular filtration pressure, and an increase in eGFR from the last value on treatment to off-treatment follow-up. Because the model requires a linear change in eGFR over time, the model was applied for the prespecified maintenance phase of treatment from week 4 to the last value on treatment.

In supplementary analyses, we applied the model to the data obtained in the study phase from baseline to off-treatment follow-up.

A rapid decline in the eGFR of >3 mL/min/1.73 m^2^/y from week 4 to the last value on treatment was the primary outcome of this analysis, and this threshold has been used in other studies.^17^^,^^18^ A more stringent definition of rapid progression, as defined according to National Kidney Foundation–Kidney Disease Outcomes Quality Initiative (NKF-KDOQI) guidelines is an individual rate of decline in eGFR >5 mL/min/1.73 m^2^/y,^19^ which was explored as a secondary outcome.

The incidence of AKI (from study drug start until study drug stop plus 7 days) was assessed among patients with and without rapid eGFR decline based on definitions of >3 mL/min/1.73 m^2^/y and >5 mL/min/1.73 m^2^/y between week 4 to last value on treatment. AKI was defined based on the respective preferred term using the medical dictionary for regulatory activities version 18.0.

### Statistical Analysis

Analyses were performed in all patients who had received at least 1 dose of a study drug. The random intercept random coefficient model to determine annualized change in eGFR included effects for treatment, baseline body mass index (BMI) and region as fixed effects, and baseline glycated hemoglobin (HbA1c), time and interaction of treatment-by-time as linear covariates. Intercepts and slopes over time were allowed to vary randomly between patients by including the patient and time as random effects. For the subgroup analysis, the models additionally included the fixed factor for the subgroup and terms for the treatment by subgroup interaction and treatment by subgroup by time interaction.

Results were expressed as an annualized average change in eGFR over the respective study period. Histogram plots of resulting individual eGFR slopes were provided by treatment arm. The eGFR slopes were separately calculated for the study periods: from baseline to follow-up (planned to be ∼30 days after cessation of treatment) and for the maintenance phase of treatment: from week 4 to last value on treatment, each requiring at least 2 measurements per patient. For the latter study period, only on-treatment data before stopping the blinded study drug and obtained before any new antidiabetic medication intake or insulin dose change was used.

Based on their estimated individual annual eGFR slopes, patients were categorized into those with rapid decline or not. Logistic regression analysis was subsequently used to investigate the differences in the odds of a rapid decline in eGFR between the empagliflozin and placebo groups, with adjustment for treatment, age, sex (categorized as male or female) baseline BMI category, baseline HbA1c category, baseline eGFR category, and geographic region.

Based on the random intercept random coefficient model and using the resulting individual participants’ intercepts and slopes we estimated the individual time to projected kidney failure (defined as the time of first reaching an eGFR value of ≤10 mL/min/1.73 m^2^, which is maintained) if a patient does not die. Therefore, we applied the model to on-treatment data from week 4 onwards and extrapolated the eGFR course, conditioning on linearity, until 15 years from baseline. Time to projected kidney failure was determined by the individual intercept and slope for participants who reached it within 15 years, while participants who did not reach it were censored at 15 years. Any death in participants within that 15-year time frame was not accounted for as a death-prediction was not performed. Treatment group differences in the risk of time to projected kidney failure were assessed using Kaplan Meier estimates and a Cox proportional hazards model with treatment, age, sex, baseline BMI category, baseline HbA1c category, baseline eGFR category, and geographic region as factors.

All analyses were performed on a nominal 2-sided α = 0·05 without adjustment for multiplicity. Additional statistical information is included in Item S1.

## Results

### Participants

Data were available for 6,967 participants (99.2% of the overall study population, Table S1) to determine the annual eGFR change from baseline to follow-up and for 5,970 participants from week 4 to the last value on treatment (Table 1). Among these, the proportion of participants with each category of baseline eGFR and urine albumin-to-creatinine ratio (UACR) aligned closely with those in the overall population (N = 7,020).^20^ In addition, 80.7% of participants overall were taking angiotensin-converting enzyme inhibitors or angiotensin-receptor blockers at baseline. Participants experiencing an eGFR decline of >3 mL/min/1.73 m^2^/y generally reported having a higher baseline eGFR and UACR as compared with participants with an annual eGFR decline of ≤3 mL/min/1.73 m^2^/y (Table 1). The median treatment duration was 2.6 years, and the median observation time was 3.1 years.^20^

**Table 1:** Baseline characteristics

Characteristic	Annual eGFR Decline in Study Period Week 4 to Last Value on Treatment			
	≤3 mL/min/1.73 m^2^/y		>3 mL/min/1.73 m^2^/y	
**Parameter, mean** **±** **SD or n (%)**	Placebo (n = 1,785)	Empagliflozin (n = 3,863)	Placebo (n = 188)	Empagliflozin (n = 134)
**Male**	1,305 (73.1)	2,762 (71.5)	125 (66.5)	90 (67.2)
**Age (y)**	63.4 ± 8.7	63.1 ± 8.5	61.9 ± 8.5	60.2 ± 8.8
**eGFR (mL/min/1.73** **m**^**2**^**), mean** **±** **SD**	73.58 ± 20.93	74.00 ± 20.82	80.02 ± 22.45	86.21 ± 23.10
**eGFR category (mL/min/1.73 m**^**2**^**), n (%)**				
≥90	360 (20.2)	829 (21.5)	64 (34.0)	56 (41.8)
60 to <90	959 (53.7)	2,048 (53.0)	95 (50.5)	64 (47.8)
45 to <60	319 (17.9)	703 (18.2)	18 (9.6)	10 (7.5)
30 to <45	141 (7.9)	269 (7.0)	11 (5.9)	4 (3.0)
<30	6 (0.3)	14 (0.4)	0	0
**UACR (mg/g), median (Q1, Q3)**	16.80 (6.19-67.18)^b^	16.80 (6.19-64.53)^c^	28.73 (7.07-201.55)^a^	24.75 (9.72-209.51)^d^
**UACR category (mg/g)**				
<30	1,079 (60.4)	2,355 (61.0)	96 (51.1)	69 (51.5)
30-300	521 (29.2)	1,091 (28.2)	50 (26.6)	37 (27.6)
>300	173 (9.7)	375 (9.7)	40 (21.3)	27 (20.1)
**Background medications**				
ACEi/ARB	1,434 (80.3)	3,126 (80.9)	150 (79.8)	108 (80.6)

*Note:* eGFR was assessed by Modification of Diet in Renal Disease Study equation.

Abbreviations: ACEi, angiotensin-converting enzyme inhibitor; ARB, angiotensin-receptor blocker; SD, standard deviation; UACR, urine albumin-to-creatinine ratio.

a n = 186.

b n = 1,773.

c n = 3,821.

d n = 133.

### Change in Kidney Function Over Time, as Measured by the Average Rate of Change in eGFR

**Figure 1:**
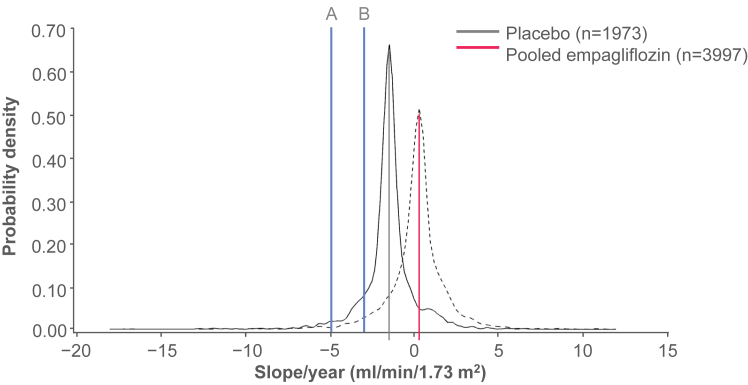
Distribution of individual eGFR changes per year in the overall population, from week 4 to last value on treatment, for placebo versus empagliflozin pooled doses. The solid blue vertical lines indicate the thresholds for an annual eGFR decline of (A) >5 mL/min/1.73 m^2^ and (B) >3 mL/min/1.73 m^2^. Patients in either treatment group with individual eGFR changes per year that appear to the left of line B are defined as having a rapid eGFR decline of >3 mL/min/1.73 m^2^/y. Similarly, patients in either treatment group with individual eGFR changes per year that appear to the left of line A are defined as having a rapid eGFR decline of >5 mL/min/1.73 m^2^/y. Adapted with permission from Wanner et al.^6^ eGFR, estimated glomerular filtration rate; LVOT, last value on treatment.

**Figure 2:**
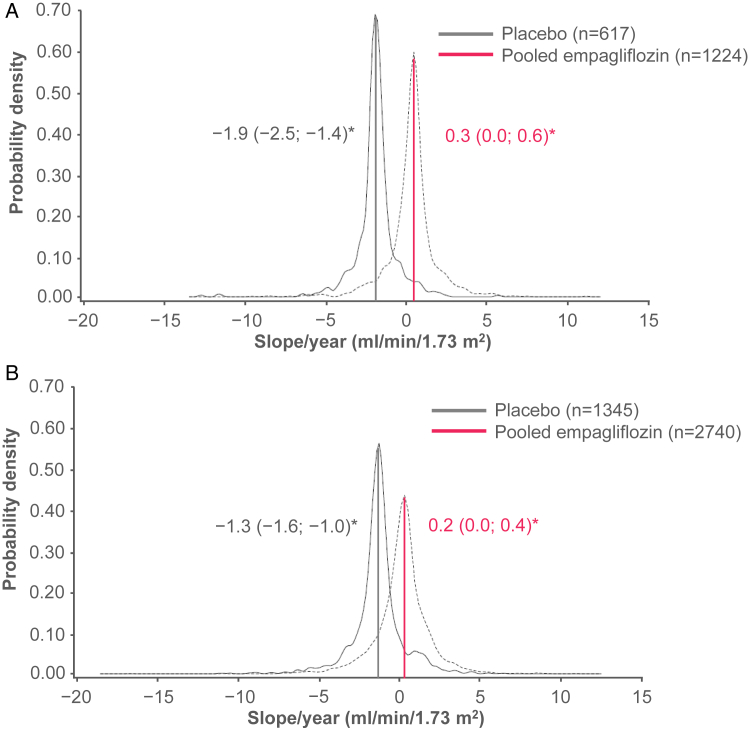
Distribution of individual eGFR changes per year and average (95% CI) eGFR change per year, from week 4 to last value on treatment, by presence or absence of prevalent CKD (eGFR of <60 mL/min/1.73 m^2^ or UACR of >300 mg/g). (A) With prevalent CKD. (B) Without prevalent CKD. CI, confidence interval; CKD, chronic kidney disease; eGFR, estimated glomerular filtration rate; UACR, urine albumin-to-creatinine ratio. ^a^Adjusted mean (95% CI) eGFR change per year per treatment group.

The average rate of eGFR change for the maintenance phase of treatment (from week 4 to last value on treatment) per treatment group and across subgroups has been reported previously: annual adjusted change in the mean eGFR of 0.2 mL/min/1.73 m^2^/y (95% confidence interval [CI], 0.1-0.4) in the empagliflozin group and −1.5 mL/min/1.73 m^2^/y (95% CI, −1.7 to −1.2) in the placebo group (*P* < 0.001 for empagliflozin vs placebo) (Fig 1) with consistency of the individual doses with the pooled analyses.^16^ The benefits of empagliflozin on the change in the eGFR were also observed at all categories of baseline albuminuria (Fig S1), all levels of baseline eGFR (Fig S2), and by participants with and without prevalent CKD (reduced eGFR or macroalbuminuria) (Fig 2A and B, respectively).

For the supplementary analysis, over the whole study period (from baseline to follow-up) in participants in the placebo arm, the eGFR declined at an average rate of −1.8 mL/min/1.73 m^2^/y (95% CI, −2.0 to −1.6; Fig S3). By contrast, the eGFR declined at an average rate of −0.3 mL/min/1.73 m^2^/y (95% CI, −0.4 to −0.1; Fig S3) in participants receiving empagliflozin. The benefits of empagliflozin on the change in the eGFR were also observed at all categories of baseline albuminuria (Fig S4), all levels of baseline eGFR (Fig S5), and including by participants with and without prevalent CKD (Fig S6).

### The Frequency of Rapid Decline in eGFR

**Figure 3:**
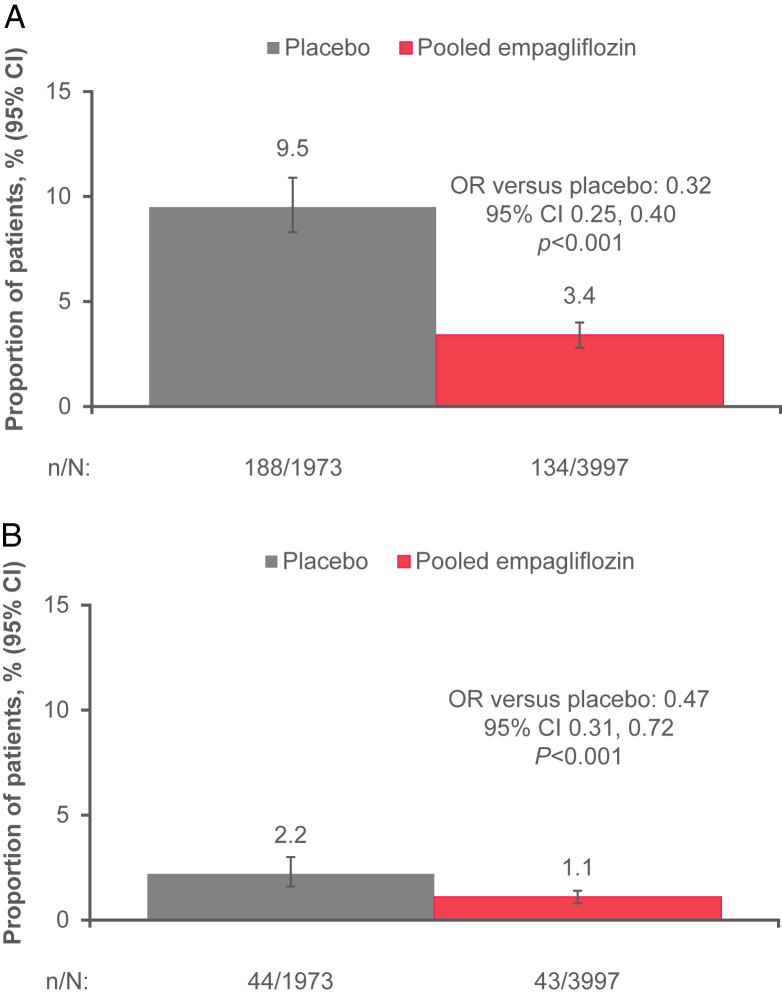
Proportion of patients with a rapid decline in eGFR of (A) >3 mL/min/1.73 m^2^/y and (B) >5 mL/min/1.73 m^2^/y, from week 4 to last value on treatment. OR based on logistic regression analysis including factors for treatment, sex, baseline BMI category, baseline HbA1c category, baseline eGFR category, geographic region, and age. The eGFR assessed by MDRD Study equation. Wilson CI for proportion. BMI, body mass index; CI, confidence interval eGFR, estimated glomerular filtration rate; HbA1c, glycated hemoglobin; LVOT, last value on treatment; MDRD, Modification of Diet in Renal Disease; OR, odds ratio.

Using the definition of eGFR decline of >3 mL/min/1.73 m^2^ per year and considering data only for the maintenance phase of treatment (week 4 to last value on treatment), in participants receiving placebo 188 (9.5%) experienced a rapid decline in kidney function compared with only 134 (3.4%) patients treated with empagliflozin (odds ratio [OR], 0.32; 95% CI, 0.25-0.40; *P* < 0.001) (Fig 3A). In patients with a decline in the eGFR of >5 mL/min/1.73 m^2^/y, 43 (1.1%) empagliflozin-treated patients versus 44 (2.2%) placebo-treated patients experienced a rapid decline in eGFR between week 4 and last value on treatment (OR, 0.47; 95% CI, 0.31-0.72; *P* < 0.001) (Fig 3B). Similarly, for the supplementary analysis, empagliflozin reduced the odds of an eGFR decline of >3 mL/min/1.73 m^2^/y over the whole study period (from baseline to follow-up) (OR, 0.35; 95% CI, 0.31-0.40; *P* < 0.001; Fig S7A) and of an eGFR decline of >5 mL/min/1.73 m^2^/y (OR, 0.33; 95% CI, 0.26-0.41; *P* < 0.001; Fig S7B). The OR for empagliflozin pooled versus placebo for a rapid decliner (>3 mL/min/1.73 m^2^/y) from week 4 to last value on treatment, by baseline eGFR and UACR, is shown in Fig S8.

### Time to Projected Kidney Failure

**Figure 4:**
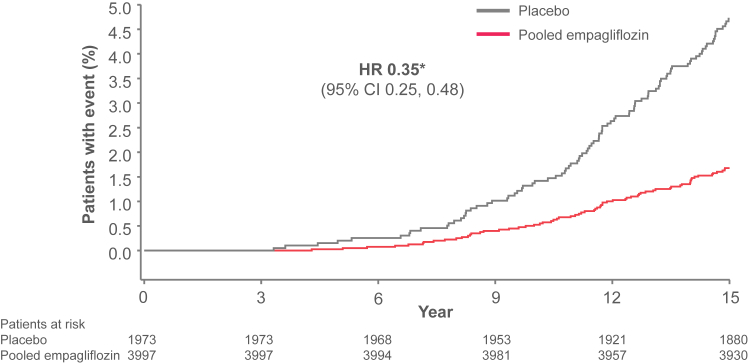
Time to projected kidney failure (defined as estimated eGFR of ≤10 mL/min/1.73 m^2^) up to 15 years. Based on the random intercept random coefficient model, applied to on-treatment data from week 4 onwards. Patients who were estimated to reach projected kidney failure of >15 years from baseline or not at all were considered censored at 15 years. Kaplan-Meier estimates. eGFR, estimated glomerular filtration rate. ^a^Hazard ratio (95% confidence interval) based on Cox regression model; *P* < 0.001.

After 15 years of follow-up 4.7% of the placebo-treated patients and 1.7% of the empagliflozin-treated patients were projected to experience end-stage kidney disease (defined as an eGFR of ≤10 mL/min/1.73 m^2^ that is maintained) based on the estimated individual patients’ intercepts and eGFR slopes and their extrapolation, assuming a patient not to die. The risk of projected kidney failure up to 15 years was reduced with empagliflozin (hazard ratio, 0.35; 95% CI, 0.25-0.48; *P* < 0.001) (Fig 4).

### Incidence of AKI

Among the patients with an annual eGFR decline of >3 mL/min/1.73 m^2^/y between week 4 and last value on treatment, AKI was reported in no patients in the placebo group and only 1 patient in the empagliflozin group; the same was seen for an eGFR decline of >5 mL/min/1.73 m^2^/y (Table S2).

## Discussion

In this post hoc analysis from the EMPA-REG OUTCOME trial, treatment with empagliflozin compared with placebo over a median of 2.6 years’ exposure to study drug was associated with a significant two-thirds reduction in the odds of experiencing a rapid decline in eGFR (>3 mL/min/1.73 m^2^/y). A comparable reduction in odds was seen using a threshold level of eGFR decline of >5 mL/min/1.73 m^2^/y.

In recent years, a number of large placebo-controlled outcome studies have demonstrated that treatment with SGLT2 inhibitors versus placebo leads to a lower risk of kidney outcomes and progression of kidney disease. These studies have included EMPA-REG OUTCOME^6^^,^^20^^,^^21^ EMPEROR-Reduced and EMPEROR-Preserved,^22^^,^^23^ CANVAS,^7^^,^^24^ CREDENCE,^11^ DECLARE-TIMI 58,^8^^,^^25^ and DAPA-HF.^26^ DAPA-CKD studied the effects of dapagliflozin on CV and kidney events in patients with CKD (also with and without T2DM).^27^ Of importance, the trial was halted early because of the overwhelming efficacy benefits of dapagliflozin.^10^^,^^28^, ^29^, ^30^ The recently finished EMPA-KIDNEY trial,^31^^,^^32^ which compared treatment with empagliflozin versus placebo in more than 6,000 people with CKD with or without diabetes, was stopped early because of a clear positive efficacy benefit with empagliflozin.^33^ The trial demonstrated that empagliflozin versus placebo significantly reduced the risk of the primary outcome (kidney disease progression or CV death) by 28% (HR, 0.72; 95% CI, 0.64-0.82; *P* < 0.001).

In EMPA-REG OUTCOME, empagliflozin reduced the risk of doubling of serum creatinine level accompanied by an eGFR of ≤45 mL/min/1.73 m^2^ (HR, 0.56 [95% CI, 0.39-0.79]).^6^ We show in the current exploratory post hoc analysis that the risk of rapid decline in eGFR, defined as an annualized decline of >3 mL/min/1.73 m^2^, was also markedly reduced in participants receiving empagliflozin. This effect was consistent with results obtained when the reversible acute decline (following initiation) and increase (following discontinuation) in eGFR associated with SGLT2 inhibition was included. Similar findings have been reported with dapagliflozin in a subgroup analysis from the DECLARE-TIMI 58 trial.^34^ As it is difficult to establish methodologically rapid kidney function decline, considering an evaluation lasting less than 3-6 months, especially when there are additional observations following that timeframe, we decided to explore the study phase from week 4 to the last value on treatment and the study phase from baseline to follow-up in supplementary analyses. The number of rapid decliners in the latter study period is increased in both treatment groups as compared with the study period from week 4 to last value on treatment, whereas the reduction in odds of rapid decline with empagliflozin versus placebo is comparable across both study periods.

Because very low rates of kidney failure were observed in EMPA-REG OUTCOME, we decided to estimate the time to projected kidney failure based on individual participants’ eGFR intercepts and slopes by extrapolation, conditioning on linearity of eGFR change over time. This expands beyond the study observation time and may not exactly reflect the often nonlinear course of disease progression. Furthermore, we assume that a patient does not die before projected kidney failure, so any differences in the mortality between the groups may have not been accounted for. Based on those assumptions, empagliflozin appeared to reduce the risk of projected kidney failure (defined as an eGFR of ≤10 mL/min/1.73 m^2^ that is maintained) up to 15 years.

In the current analysis, the incidence of AKI was both low and comparable across treatment groups and not increased in patients with an eGFR decline of >3 mL/min/1.73 m^2^/y and >5 mL/min/1.73 m^2^/y versus without. This suggests that rapid decliner status seems to not predispose to AKI.

A number of different mechanisms by which SGLT2 inhibition may preserve kidney function in patients with diabetes have been proposed, including direct actions against diabetic kidney disease.^35^ However, in the current analysis, in addition to the lower incidence of rapidly worsening kidney function with empagliflozin, observed in participants with prevalent CKD at baseline, the distribution of eGFR change was uniformly shifted to the right in patients treated with empagliflozin, meaning that at the same time that fewer participants experienced a rapid decline in eGFR, more patients experienced improvements in their kidney function. If empagliflozin simply modulated pathophysiologic factors associated with eGFR decline (ie, had a proportional effect), while the lower tail of the distribution may be curtailed, there is no reason that the whole distribution curve would shift to the right. Consistent with this observation, even in participants without CKD (defined as those without a reduced eGFR and the absence of macroalbuminuria) uniform modulation in the distribution of eGFR change was observed in participants treated with empagliflozin.

Taken together, these findings suggest that, at least in the short-term, empagliflozin may not just be acting on diabetic kidney disease or CKD,^33^ but also may be protecting kidney function more generally, even in the absence of established signs of CKD. Potential mechanisms for renoprotection include reductions in intraglomerular pressure, arterial stiffness, and neurohormonal signaling, and reduced hypoxia and oxidative stress in the proximal tubule contributing to nephron dropout.^36^, ^37^, ^38^, ^39^, ^40^, ^41^, ^42^ Other mechanisms might be related to the beneficial effect of empagliflozin, such as a decrease in uric acid, the “super-fuel” hypothesis, suggesting that a switch to ketone bodies might lead to a more efficient kidney work. However, the search for the mechanistic is beyond the scope of this study.

Some studies have reported that SGLT2 inhibition may have the potential to attenuate hyperfiltration.^36^^,^^42^, ^43^, ^44^ Indeed, SGLT2 inhibition is associated with an initial decrease in eGFR of 3-5 mL/min/1.73 m^2^ shortly after treatment initiation,^6^^,^^16^^,^^45^^,^^46^ which may lead to an underestimation of the true effect of SGLT2 inhibition to stabilize kidney function over a longer period of time. In our analysis, the relative effect of SGLT2 inhibition to stabilize kidney function was comparable when excluding data from the first 4 weeks of the study. Similar kidney benefits were also observed regardless of baseline eGFR and whether or not the eGFR declined after initiation of empagliflozin.^21^

There are limitations to our findings. Kidney outcomes were not the primary outcome of the EMPA-REG OUTCOME trial, and therefore the present analysis is post-hoc and primarily exploratory in nature. Generalization of the findings to a broader patient population with CKD also has limitations because only patients with T2DM were studied, not necessarily with CKD at baseline. Furthermore, the eGFR status before randomization was not known. Therefore, although we believe there is likely to be a causal effect of empagliflozin in reducing the risk of a rapid eGFR decline, it is possible that some patients already had a “rapid decliner” phenotype at baseline, rather than de novo development of such a phenotype during the study, and that fewer patients had this phenotype in the empagliflozin arm. The results of EMPA-KIDNEY have provided greater insight of the benefits of SGLT2 inhibitors in patients with CKD with or without diabetes, including those with CKD without albuminuria. Analyses of AKI by rapid decline were limited by patient groups being selected based on postrandomization information and may therefore not constitute comparable groups which precludes making definitive conclusions about treatment effects. Acute kidney injury was defined based on adverse events registered corresponding to AKI. Although adverse events were asked to be captured during every patient visit, there were no specific criteria provided to the investigators and such a definition is restrictive.

In conclusion, among patients with T2DM with cardiovascular disease, fewer patients treated with empagliflozin experienced a rapid decline in kidney function of >3 mL/min/1.73 m^2^/y when compared with patients receiving placebo during the trial on-treatment period. The distribution of individual annualized eGFR changes was shifted to the right in empagliflozin-treated patients, which would suggest a lower risk of rapid decline in the eGFR compared with placebo which, in turn, would lead to a reduced risk for projected kidney failure over an extrapolated period of 15 years of follow-up.

## Supplementary Material

Supplementary File (PDF) Figure S1-S8; Item S1; Table S1-S2.
