# Supplementary material for: Empagliflozin and Rapid Kidney Function Decline Incidence in Type 2 Diabetes: An Exploratory Analysis From the EMPA-REG OUTCOME Trial
Source: Kidney Med. 2023 Dec 18;6(3):100783. doi: 10.1016/j.xkme.2023.100783 (PMC10900108; doi:10.1016/j.xkme.2023.100783)
Supplement: Supplementary File (PDF) — Figure S1-S8; Item S1; Table S1-S2. [file mmc1.pdf]

**Figure S1.** Distribution of individual eGFR changes per year and average (95% CI) eGFR change per year, from week 4 to last value on treatment, by baseline UACR. A, normoalbuminuria (UACR <30 mg/g); B, microalbuminuria (UACR 30 to 300 mg/g); C, macroalbuminuria (UACR >300 mg/g). *Adjusted mean (95% CI) eGFR change per year per treatment group. CI, confidence interval; eGFR, estimated glomerular filtration rate; UACR, urine albumin-to-creatinine ratio.

A

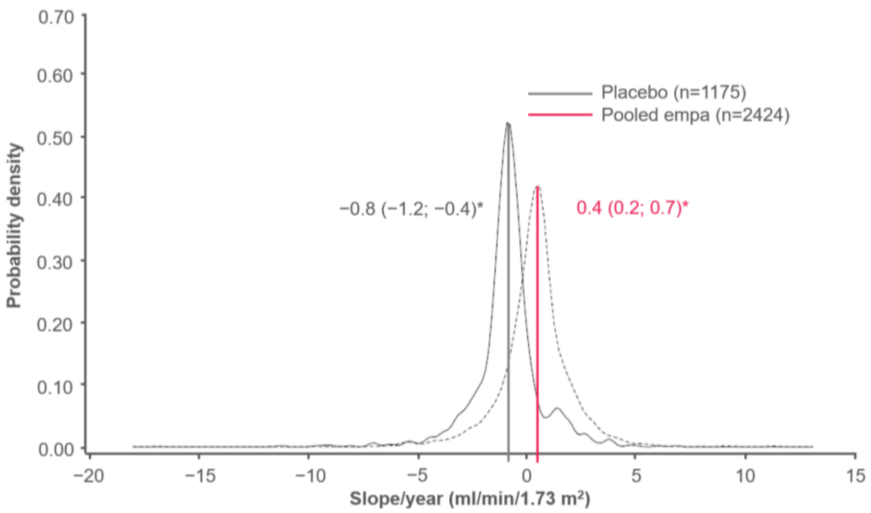


B

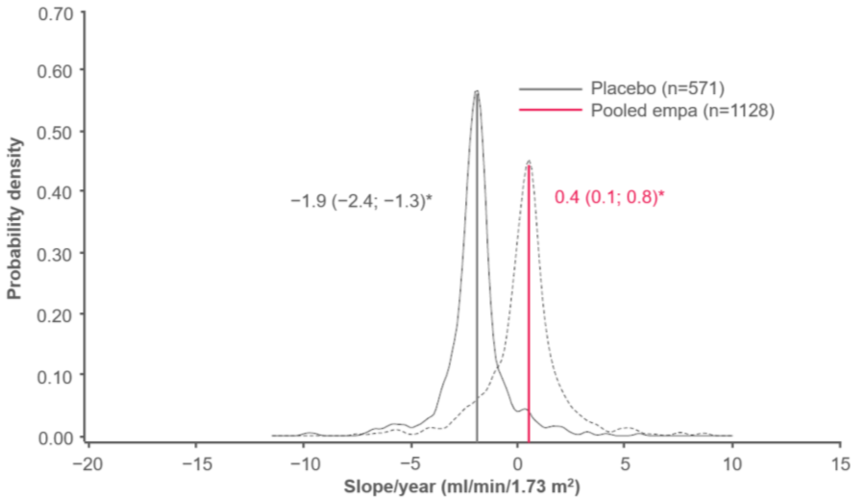

C

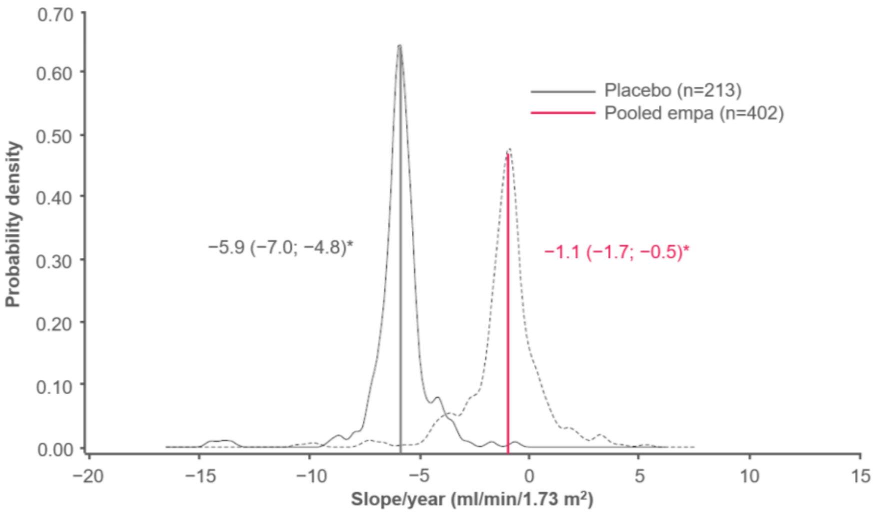

0.70
0.60
0.50
0.40
0.30
0.20
0.10
0.00
Probability density
Placebo (n=213)
Pooled empa (n=402)
−5.9 (−7.0; −4.8)*
−1.1 (−1.7; −0.5)*
−20
−15
−10
−5
0
5
10
15
Slope/year (ml/min/1.73 m²)

**Figure S2.** Distribution of individual eGFR changes per year and average (95% CI) eGFR change per year, from week 4 to last value on treatment, by baseline eGFR. A, eGFR ≥90 ml/min/1.73 $m^2$; B, eGFR 60 to <90 ml/min/1.73 $m^2$; C, eGFR 45 to <60 ml/min/1.73 $m^2$; D, eGFR <45 ml/min/1.73 $m^2$. *Adjusted mean (95% CI) eGFR change per year per treatment group. CI, confidence interval; eGFR, estimated glomerular filtration rate.

A B

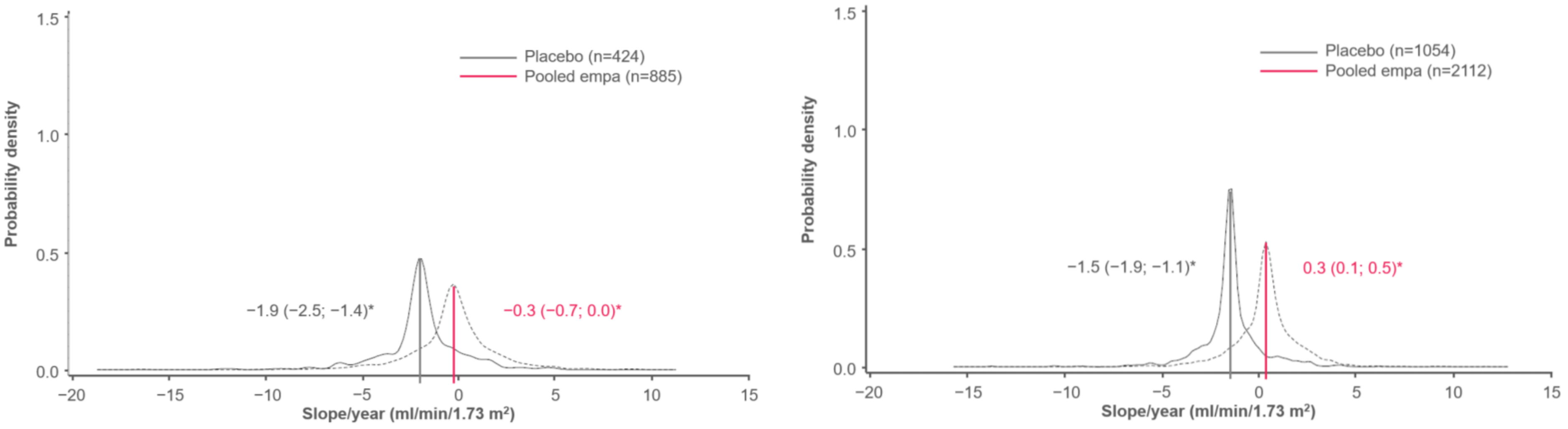

C

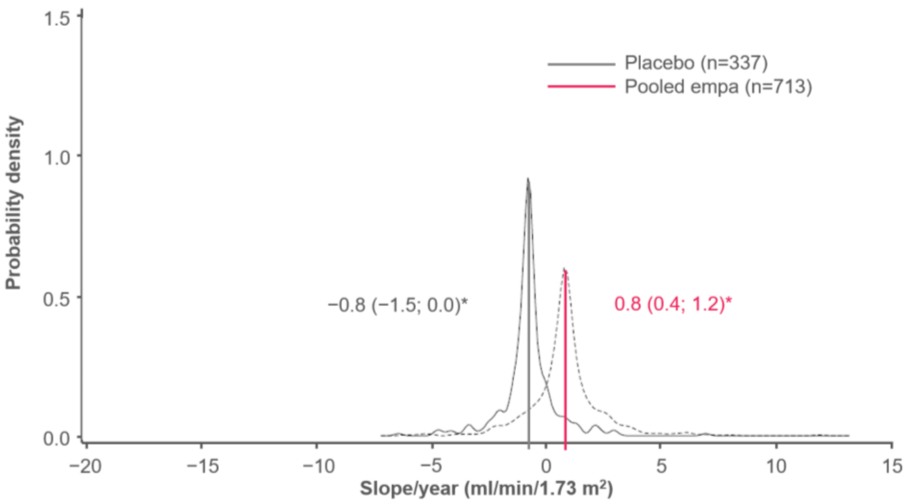
Placebo (n=337)
Pooled empa (n=713)
Probability density
−0.8 (−1.5; 0.0)*
0.8 (0.4; 1.2)*
Slope/year (ml/min/1.73 m²)

D

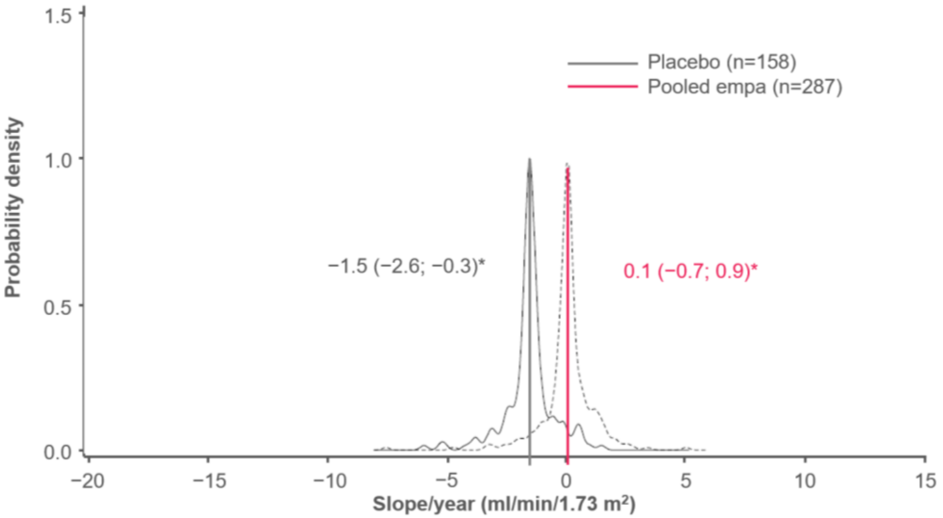
Placebo (n=158)
Pooled empa (n=287)
Probability density
−1.5 (−2.6; −0.3)*
0.1 (−0.7; 0.9)*
Slope/year (ml/min/1.73 m²)

**Figure S3.** Distribution of individual eGFR changes per year and average (95% CI) eGFR change per year in the overall population, from baseline to follow-up, per treatment group. *Adjusted mean (95% CI) eGFR change per year. CI, confidence interval; eGFR, estimated glomerular filtration rate.

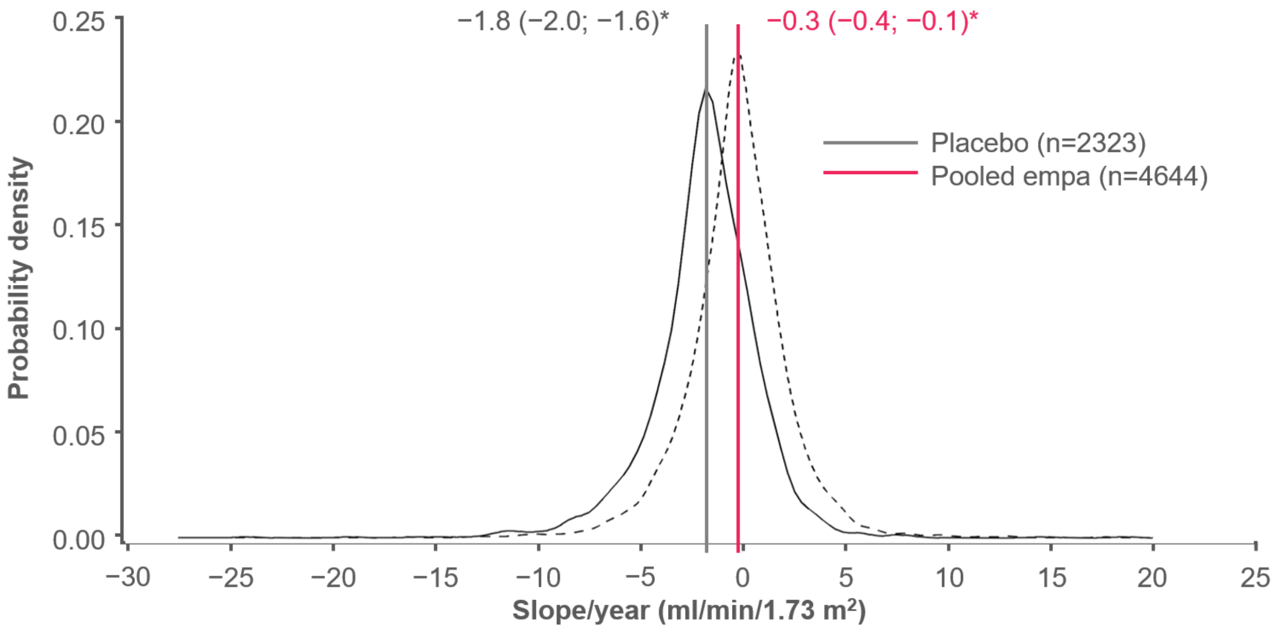

**Figure S4.** Distribution of individual eGFR changes per year and average (95% CI) eGFR change per year, from baseline to follow-up, by baseline UACR. A, normoalbuminuria (UACR <30 mg/g); B, microalbuminuria (UACR 30 to 300 mg/g); C, macroalbuminuria (UACR >300 mg/g). *Adjusted mean (95% CI) eGFR change per year per treatment group. CI, confidence interval; eGFR, estimated glomerular filtration rate; UACR, urine albumin-to-creatinine ratio.

A

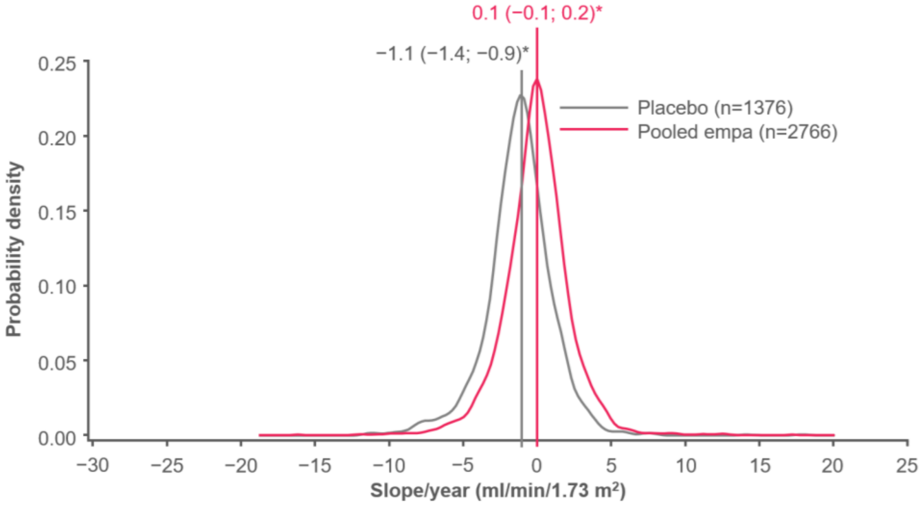


B

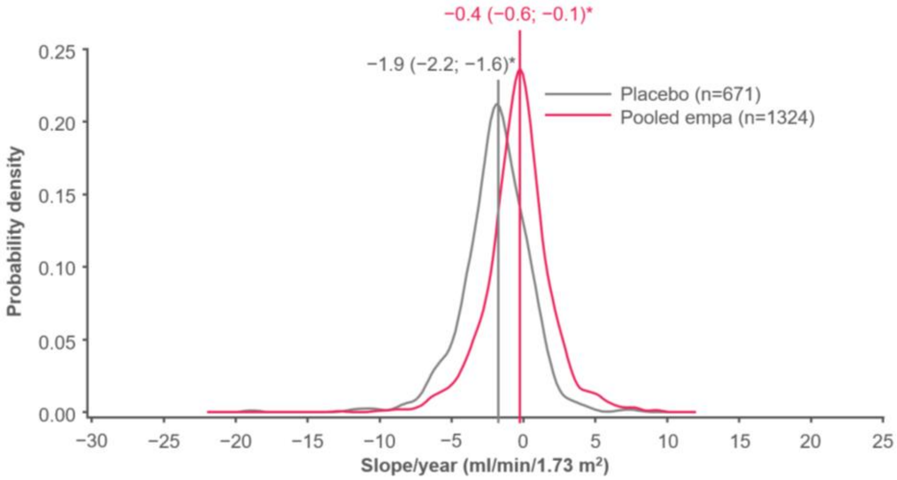

C

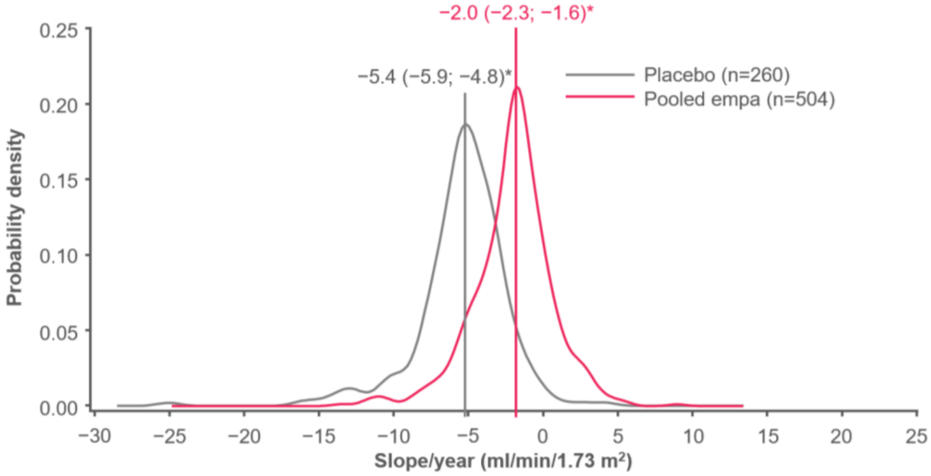
−2.0 (−2.3; −1.6)*
−5.4 (−5.9; −4.8)*
Placebo (n=260)
Pooled empa (n=504)
Probability density
0.25
0.20
0.15
0.10
0.05
0.00
−30
−25
−20
−15
−10
−5
0
5
10
15
20
25
Slope/year (ml/min/1.73 m²)

**Figure S5.** Distribution of individual eGFR changes per year and average (95% CI) eGFR change per year, from baseline to follow-up, by baseline eGFR. A, eGFR ≥90 ml/min/1.73 $m^2$; B, eGFR 60 to <90 ml/min/1.73 $m^2$; C, eGFR 45 to <60 ml/min/1.73 $m^2$; D, eGFR <45 ml/min/1.73 $m^2$. *Adjusted mean (95% CI) per year per treatment group. CI, confidence interval; eGFR, estimated glomerular filtration rate.

A

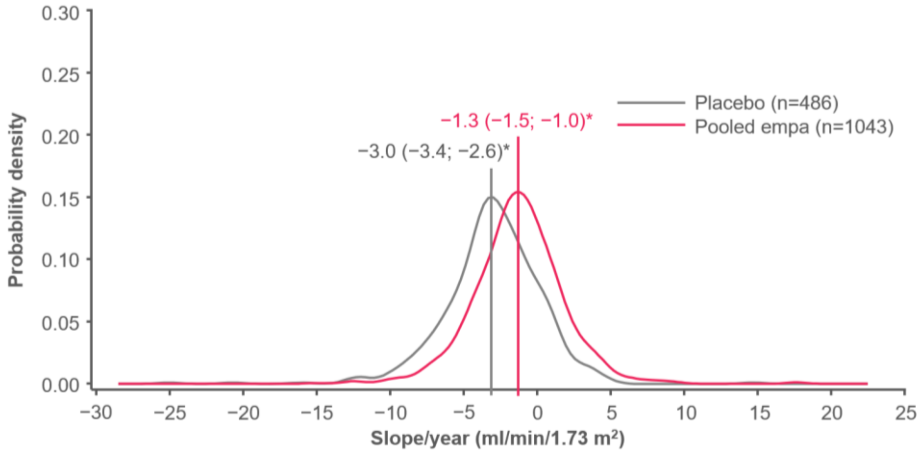


B

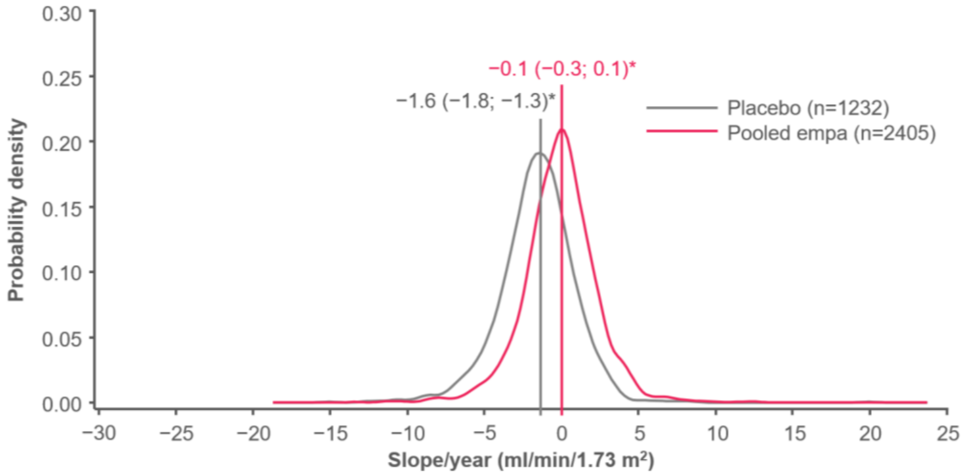

C

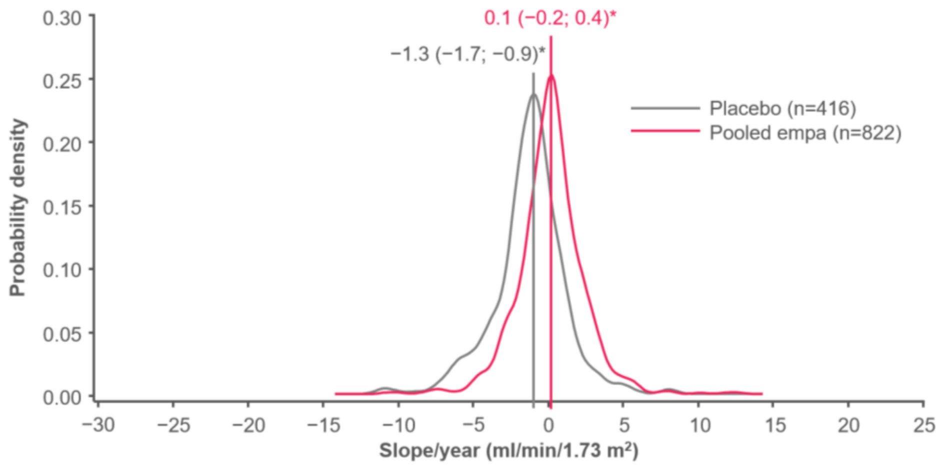
0.1 (−0.2; 0.4)*
−1.3 (−1.7; −0.9)*
Placebo (n=416)
Pooled empa (n=822)
Probability density
0.30
0.25
0.20
0.15
0.10
0.05
0.00
−30
−25
−20
−15
−10
−5
0
5
10
15
20
25
Slope/year (ml/min/1.73 m²)

D

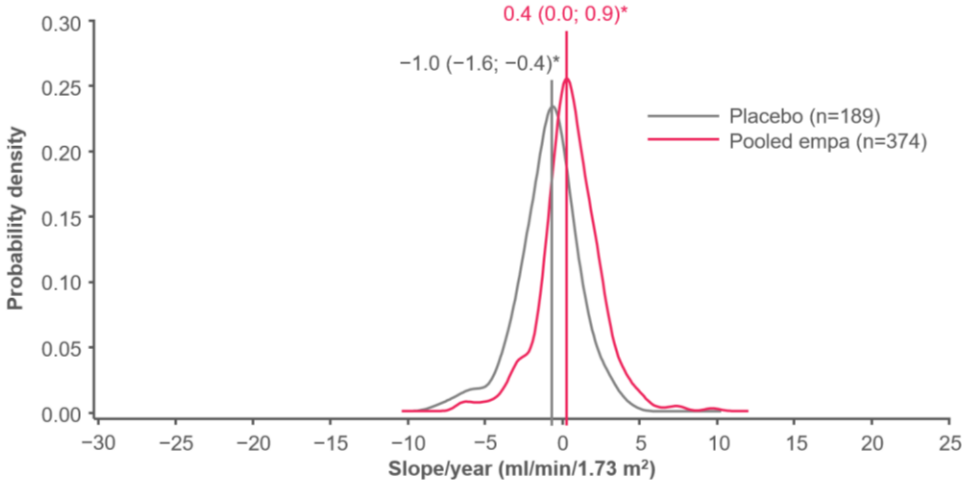
0.4 (0.0; 0.9)*
−1.0 (−1.6; −0.4)*
Placebo (n=189)
Pooled empa (n=374)
Probability density
0.30
0.25
0.20
0.15
0.10
0.05
0.00
−30
−25
−20
−15
−10
−5
0
5
10
15
20
25
Slope/year (ml/min/1.73 m²)

**Figure S6.** Distribution of individual eGFR changes per year and average (95% CI) eGFR change per year, from baseline to follow-up, by presence or absence of prevalent CKD (eGFR <60 ml/min/1.73 m$^2$ and/or UACR >300 mg/g). A, with prevalent CKD; B, without prevalent CKD. *Adjusted mean (95% CI) per year per treatment group. CI, confidence interval; CKD, chronic kidney disease; eGFR, estimated glomerular filtration rate; UACR, urine albumin-to-creatinine ratio.

A

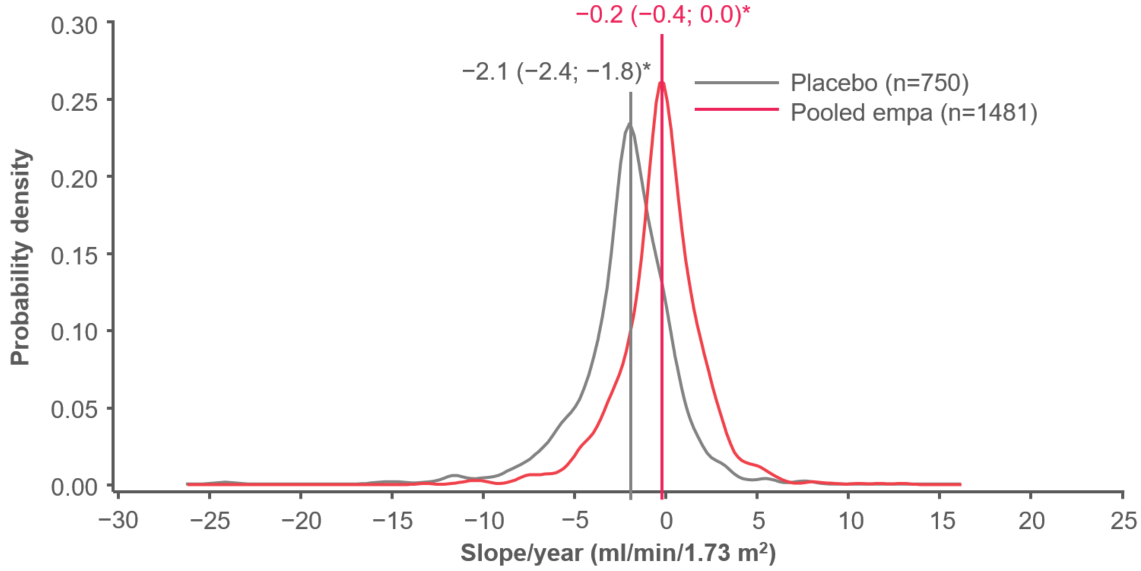


B

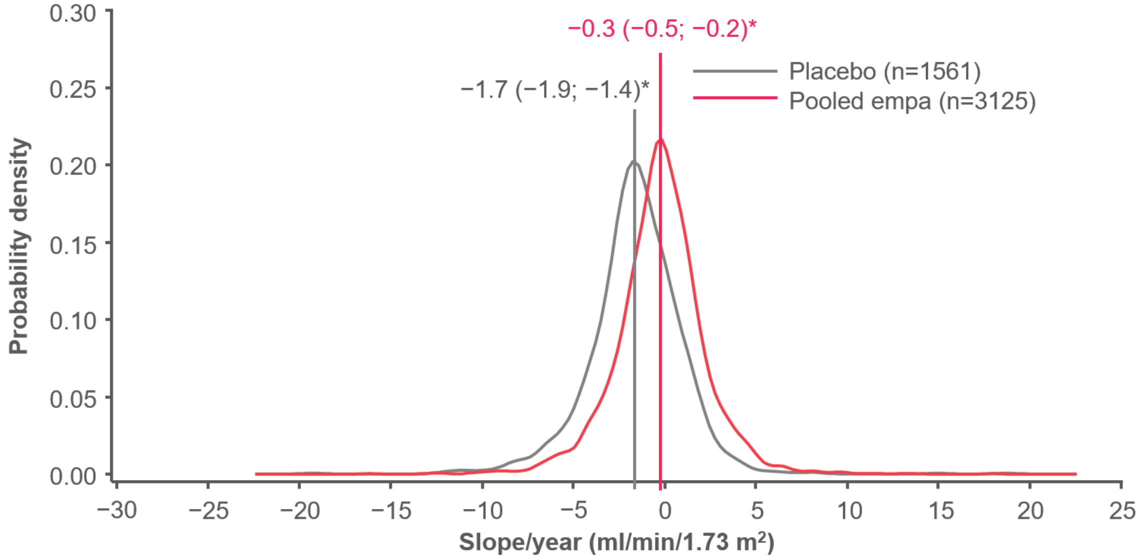

**Figure S7.** Proportion of patients with a rapid decline in eGFR of (A) >3 ml/min/1.73m$^2$ per year and (B) >5 ml/min/1.73m$^2$ per year, from baseline to follow-up. OR based on logistic regression analysis including factors for treatment, sex, baseline BMI category, baseline HbA1c category, baseline eGFR category, geographical region and age. eGFR assessed by MDRD formula. Wilson CI for proportion. BMI, body mass index; CI, confidence interval; eGFR, estimated glomerular filtration rate; HbA1c, glycated haemoglobin; MDRD, Modification of Diet in Renal Disease; OR, odds ratio.

A

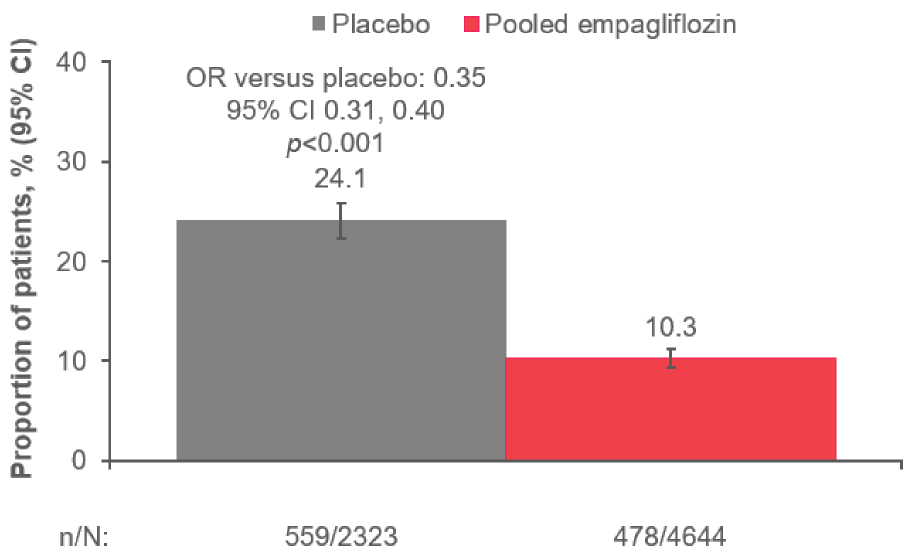

B

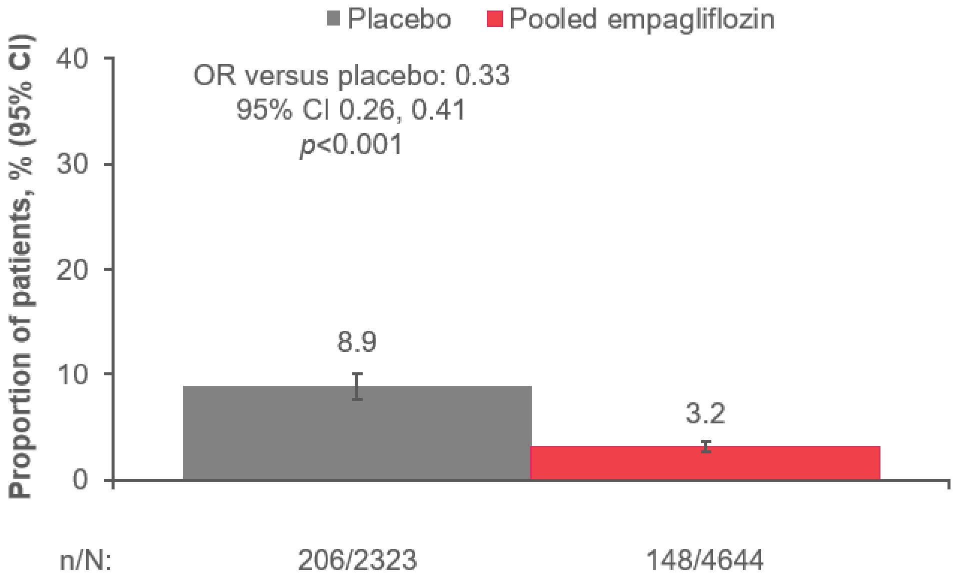
Placebo
Pooled empagliflozin
Proportion of patients, % (95% CI)
40
30
20
10
0
OR versus placebo: 0.33
95% CI 0.26, 0.41
p<0.001
8.9
3.2
n/N:
206/2323
148/4644

**Figure S8.** Odds ratio for empagliflozin pooled versus placebo for rapid decliner (>3 ml/min/1.73m$^2$ per year), from week 4 to LVOT, by baseline eGFR and UACR. Based on logistic regression analysis. CI, confidence interval; eGFR, estimated glomerular filtration rate; LVOT, last value on treatment; UACR, urine albumin-to-creatinine ratio.

| | Empagliflozin pooled n/N (%) | Placebo n/N (%) | Odds ratio (95% CI) Empa vs placebo | Interaction *p*-value |
|---|---|---|---|---|
| **eGFR, ml/min/1.73 m$^2$** | | | | |
| Overall | 134/4678 (3.4) | 188/2333 (9.5) | 0.32 (0.25, 0.40) | |
| ≥90 | 56/885 (6.3) | 64/424 (15.1) | 0.38 (0.26, 0.55) | |
| 60 to <90 | 64/2112 (3.0) | 95/1054 (9.0) | 0.31 (0.23, 0.43) | *p*=0.58 |
| 45 to <60 | 10/713 (1.4) | 18/337 (5.3) | 0.25 (0.11, 0.55) | |
| <45 | 4/287 (1.4) | 11/158 (7.0) | 0.19 (0.06, 0.61) | |
| **UACR, mg/g** | | | | |
| Normal | 69/2424 (2.8) | 96/1175 (8.2) | 0.32 (0.23, 0.44) | |
| Microalbuminuria | 37/1128 (3.3) | 50/571 (8.8) | 0.34 (0.22, 0.53) | *p*=0.94 |
| Macroalbuminuria | 27/402 (6.7) | 40/213 (18.8) | 0.30 (0.18, 0.52) | |

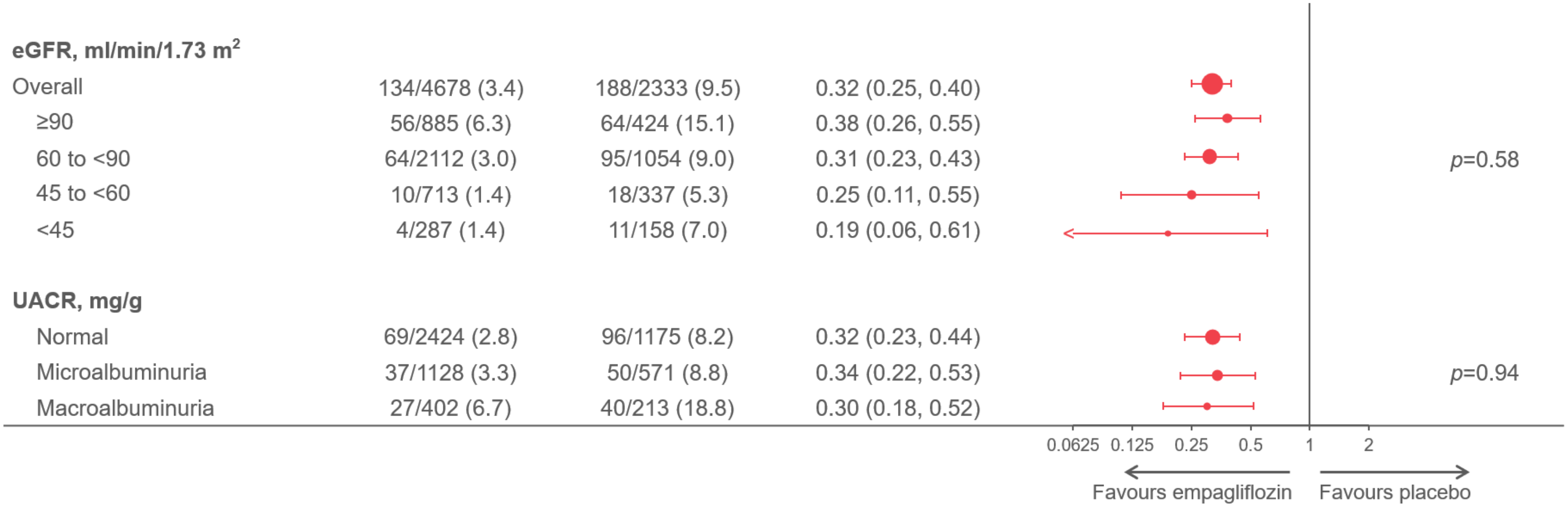

**Item S1. Supplementary Statistical Information**

For the eGFR slope analysis reported here, the patient’s average rate of change of eGFR per year was obtained using random intercept random coefficient models. Hereby the annual change in eGFR was modeled as a linear decline per treatment group. eGFR annualized changes were separately evaluated during the two pre-specified trial periods, first when patients received stable treatment with study drug (week 4 until last value on-treatment; i.e., the chronic maintenance treatment period), and second from baseline (i.e. after starting treatment) until follow-up (planned to be approximately 30 days after cessation of treatment). For the former study period, only on-treatment data before stopping the blinded study drug and obtained prior to new antidiabetic medication intake/insulin dose change was used. Results were expressed as an annualized average change in eGFR over the respective study period. Histogram plots of resulting individual patient’s average rate of change of eGFR per year were provided by treatment arm.

Further, the annualized changes in eGFR were assessed in relevant subgroups.

The random intercept random coefficient model to determine annualized change in eGFR included effects for treatment, baseline body-mass index (BMI) and region as fixed classification effects and baseline glycated haemoglobin (HbA1c), time and interaction of treatment-by-time as linear covariates. Intercepts and slopes over time were allowed to vary randomly between patients by including the patient and time as random effects. For the subgroup analysis, the models additionally included the fixed factor for the subgroup as well as terms for treatment by subgroup interaction and treatment by subgroup by time interaction. Patients were required to

provide at least 2 measurements per study period to be included in the respective analysis.

Hereby the available eGFR measurements as obtained at planned study visits until the earliest as of individual study termination or death were included in the analyses, irrespective of occurrence or timing of renal replacement therapy. (As previously reported[6], the initiation of renal replacement therapy occurred in 13 of 4687 patients (0.3%) in the empagliflozin group and in 14 of 2333 (0.6%) in the placebo group only. There were three deaths from renal disease in the empagliflozin group (0.1%) and none in the placebo group.)

Because empagliflozin was previously shown to exert an acute haemodynamic effect after start of medication and an increase in eGFR after empagliflozin treatment was stopped, the main analyses in this manuscript focus on the eGFR slope assessed during the chronic maintenance period, the study period where the linearity assumption for eGFR change is justified by the data, and results for the study period from baseline to follow-up are considered supplementary.

**Table S1.** Baseline characteristics by treatment group (overall population).

| **Characteristic*** | **Placebo (N = 2333)** | **Empagliflozin 10 mg (N = 2345)** | **Empagliflozin 25 mg (N = 2342)** | **Pooled empagliflozin (N = 4687)** |
|---|---|---|---|---|
| Age – years | 63.2 ± 8.8 | 63.0 ± 8.6) | 63.2 ± 8.6) | 63.1 ± 8.6 |
| Male – no. (%) | 1680 (72.0) | 1653 (70.5) | 1683 (71.9) | 3336 (71.2) |
| Race – no. (%) | | | | |
| White | 1678 (71.9) | 1707 (72.8) | 1696 (72.4) | 3403 (72.6) |
| Asian | 511 (21.9) | 505 (21.5) | 501 (21.4) | 1006 (21.5) |
| Black/African-American | 120 (5.1) | 119 (5.1) | 118 (5.0) | 237 (5.1) |
| Other/Missing | 24 (1.0) | 14 (0.6) | 27 (1.2) | 41 (0.9) |
| Ethnicity – no. (%) | | | | |
| Not Hispanic or Latino | 1912 (82.0) | 1909 (81.4) | 1926 (82.2) | 3835 (81.8) |
| Hispanic or Latino | 418 (17.9) | 432 (18.4) | 415 (17.7) | 847 (18.1) |
| Missing | 3 (0.1) | 4 (0.2) | 1 (<0.1) | 5 (0.1) |
| Region – no. (%) | | | | |
| Europe | 959 (41.1) | 966 (41.2) | 960 (41.0) | 1926 (41.1) |
| North America (plus Australia and New Zealand) | 462 (19.8) | 466 (19.9) | 466 (19.9) | 932 (19.9) |
| Asia | 450 (19.3) | 447 (19.1) | 450 (19.2) | 897 (19.1) |
| Latin America | 360 (15.4) | 359 (15.3) | 362 (15.5) | 721 (15.4) |
| Africa | 102 (4.4) | 107 (4.6) | 104 (4.4) | 211 (4.5) |
| Weight – kg | 86.6 ± 19.1 | 85.9 ± 18.8 | 86.5 ± 19.0 | 86.2 ± 18.9 |
| Body mass index – kg/$m^{2}$[†] | 30.7 ± 5.2 | 30.6 ± 5.2 | 30.6 ± 5.3 | 30.6 ± 5.3 |

| | | | | |
|---|---|---|---|---|
| CV risk factor – no. (%) | 2307 (98.9) | 2333 (99.5) | 2324 (99.2) | 4657 (99.4) |
| Coronary artery disease | 1763 (75.6) | 1782 (76.0) | 1763 (75.3) | 3545 (75.6) |
| Multi-vessel coronary artery disease | 1100 (47.1) | 1078 (46.0) | 1101 (47.0) | 2179 (46.5) |
| History of myocardial infarction | 1083 (46.4) | 1107 (47.2) | 1083 (46.2) | 2190 (46.7) |
| Coronary artery bypass graft | 563 (24.1) | 594 (25.3) | 581 (24.8) | 1175 (25.1) |
| History of stroke[‡] | 553 (23.7) | 535 (22.8) | 549 (23.4) | 1084 (23.1) |
| Peripheral artery disease | 479 (20.5) | 465 (19.8) | 517 (22.1) | 982 (21.0) |
| Single vessel coronary artery disease[‡] | 238 (10.2) | 258 (11.0) | 240 (10.2) | 498 (10.6) |
| Cardiac failure[§] | 244 (10.5) | 240 (10.2) | 222 (9.5) | 462 (9.9) |
| Glycated hemoglobin – %[¶] | 8.08 ± 0.84 | 8.07 ± 0.86 | 8.06 ± 0.84 | 8.07 ± 0.85 |
| Time since diagnosis of type 2 diabetes – no. (%) | | | | |
| ≤1 years | 52 (2.2) | 68 (2.9) | 60 (2.6) | 128 (2.7) |
| >1 to 5 years | 371 (15.9) | 338 (14.4) | 374 (16.0) | 712 (15.2) |
| >5 to 10 years | 571 (24.5) | 585 (24.9) | 590 (25.2) | 1175 (25.1) |
| >10 years | 1339 (57.4) | 1354 (57.7) | 1318 (56.3) | 2672 (57.0) |
| Glucose-lowering therapy – no. (%) | | | | |
| Medication taken alone or in combination | | | | |
| Metformin | 1734 (74.3) | 1729 (73.7) | 1730 (73.9) | 3459 (73.8) |
| Insulin | 1135 (48.6) | 1132 (48.3) | 1120 (47.8) | 2252 (48.0) |
| Median daily dose – IU[‖] | 52.0 | 52.5 | 54.0 | 54.0 |
| Sulfonylurea | 992 (42.5) | 985 (42.0) | 1029 (43.9) | 2014 (43.0) |
| Dipeptidyl peptidase-4 inhibitor | 267 (11.4) | 282 (12.0) | 247 (10.5) | 529 (11.3) |

| | | | | |
|---|---|---|---|---|
| Thiazolidinedione | 101 (4.3) | 96 (4.1) | 102 (4.4) | 198 (4.2) |
| Glucagon-like peptide-1 agonist | 70 (3.0) | 68 (2.9) | 58 (2.5) | 126 (2.7) |
| Monotherapy | 691 (29.6) | 704 (30.0) | 676 (28.9) | 1380 (29.4) |
| Dual therapy | 1148 (49.2) | 1110 (47.3) | 1149 (49.1) | 2259 (48.2) |
| Anti-hypertensive therapy – no. (%) | 2221 (95.2) | 2227 (95.0) | 2219 (94.7) | 4446 (94.9) |
| Angiotensin-converting enzyme inhibitors/angiotensin receptor blockers | 1868 (80.1) | 1896 (80.9) | 1902 (81.2) | 3798 (81.0) |
| Beta-blockers | 1498 (64.2) | 1530 (65.2) | 1526 (65.2) | 3056 (65.2) |
| Diuretics | 988 (42.3) | 1036 (44.2) | 1011 (43.2) | 2047 (43.7) |
| Calcium channel blockers | 788 (33.8) | 781 (33.3) | 748 (31.9) | 1529 (32.6) |
| Mineralocorticoid receptor antagonists | 136 (5.8) | 157 (6.7) | 148 (6.3) | 305 (6.5) |
| Renin inhibitors | 19 (0.8) | 16 (0.7) | 11 (0.5) | 27 (0.6) |
| Other | 191 (8.2) | 193 (8.2) | 190 (8.1) | 383 (8.2) |
| Lipid-lowering therapy – no. (%) | 1864 (79.9) | 1926 (82.1) | 1894 (80.9) | 3820 (81.5) |
| Statins | 1773 (76.0) | 1827 (77.9) | 1803 (77.0) | 3630 (77.4) |
| Fibrates | 199 (8.5) | 214 (9.1) | 217 (9.3) | 431 (9.2) |
| Ezetimibe | 81 (3.5) | 95 (4.1) | 94 (4.0) | 189 (4.0) |
| Niacin | 35 (1.5) | 56 (2.4) | 35 (1.5) | 91 (1.9) |
| Other | 175 (7.5) | 172 (7.3) | 193 (8.2) | 365 (7.8) |
| Anti-coagulants – no. (%) | 2090 (89.6) | 2098 (89.5) | 2064 (88.1) | 4162 (88.8) |
| Acetylsalicylic acid | 1927 (82.6) | 1939 (82.7) | 1937 (82.7) | 3876 (82.7) |
| Clopidogrel | 249 (10.7) | 253 (10.8) | 241 (10.3) | 494 (10.5) |
| Vitamin K antagonists | 156 (6.7) | 141 (6.0) | 125 (5.3) | 266 (5.7) |

| | | | | |
|---|---|---|---|---|
| Systolic blood pressure – mmHg | 135.8 ± 17.2 | 134.9 ± 16.8 | 135.6 ± 17.0 | 135.3 ± 16.9 |
| Diastolic blood pressure – mmHg | 76.8 ± 10.1 | 76.6 ± 9.8 | 76.6 ± 9.7 | 76.6 ± 9.7 |
| Total cholesterol – mg/dL** | 161.9 ± 43.1 | 163.7 ± 45.2 | 163.3 ± 43.2 | 163.5 ± 44.2 |
| Low density lipoprotein cholesterol – mg/dL†† | 84.9 ± 35.3 | 86.3 ± 36.7 | 85.5 ± 35.2 | 85.9 ± 36.0 |
| High density lipoprotein cholesterol – mg/dL** | 44.0 ± 11.3 | 44.7 ± 12.0 | 44.5 ± 11.8 | 44.6 ± 11.9 |
| Triglycerides – mg/dL** | 170.7 ± 121.2 | 168.4 ± 127.3 | 172.6 ± 132.0 | 170.5 ± 129.7 |
| Estimated glomerular filtration rate – mL/min/1.73m$^2$‡‡ | 73.8 ± 21.1 | 74.3 ± 21.8 | 74.0 ± 21.4 | 74.2 ± 21.6 |
| Estimated glomerular filtration rate – no. (%)‡‡ | | | | |
| ≥90 mL/min/1.73m$^2$ | 488 (20.9) | 519 (22.1) | 531 (22.7) | 1050 (22.4) |
| 60 to <90 mL/min/1.73m$^2$ | 1238 (53.1) | 1221 (52.1) | 1202 (51.3) | 2423 (51.7) |
| <60 mL/min/1.73m$^2$ | 607 (26.0) | 605 (25.8) | 607 (25.9) | 1212 (25.9) |
| Urine albumin-to-creatinine ratio – no. (%)§§ | | | | |
| <30 mg/g | 1382 (59.2) | 1405 (59.9) | 1384 (59.1) | 2789 (59.5) |
| 30 to 300 mg/g | 675 (28.9) | 645 (27.5) | 693 (29.6) | 1338 (28.5) |
| >300 mg/g | 260 (11.1) | 261 (11.1) | 248 (10.6) | 509 (10.9) |

From *New England Journal of Medicine*, Zinman B, Wanner C, Lachin JM, Fitchett D, Bluhmki E, Hantel S, Mattheus M, Devins T, Johansen OE, Woerle HJ, Broedl UC, Inzucchi SE, for the EMPA-REG OUTCOME Investigators. Empagliflozin, Cardiovascular Outcomes, and Mortality in Type 2 Diabetes, 373, 2117–2128. Copyright © 2015, Massachusetts Medical Society. Reprinted with permission from Massachusetts Medical Society.

* Plus–minus values are means ± SD.

† Body mass index is the weight in kilograms divided by the square of the height in meters.

‡ Information was not available for one patient in the placebo group.

§ Based on the narrow standard MedDRA query 'cardiac failure'.

¶ Data were available for 2333 patients in the placebo group, 2344 patients in the empagliflozin 10 mg group, 2341 patients in the empagliflozin

25 mg group.
‖ Data were not available for 18 patients in the placebo group, 10 patients in the empagliflozin 10 mg group and 14 patients in the empagliflozin
25 mg group.
** Data were available for 2309 patients in the placebo group, 2318 patients in the empagliflozin 10 mg group, 2308 patients in the empagliflozin 25 mg group. Conversion factor: 1 mg/dL = 0.02586 mmol/L for cholesterol and 1 mg/dL = 0.01129 mmol/L for triglycerides.
†† Data were available for 2309 patients in the placebo group, 2317 patients in the empagliflozin 10 mg group, 2306 patients in the empagliflozin 25 mg group. 1 mg/dL = 0.02586 mmol/L.
‡‡ Data were not available for 2 patients in the empagliflozin 25 mg group. The estimated glomerular filtration rate was calculated using the
Modification of Diet in Renal Disease formula.
§§ Data were not available for 16 patients in the placebo group, 34 patients in the empagliflozin 10 mg group, 17 patients in the empagliflozin 25
mg group.
There were no significant differences ($p<0.05$) between pooled empagliflozin and placebo based on Chi-square test for binary/categorical
variables, t-test for continuous variables, and Wilcoxon rank sum test for insulin dose.

**Table S2.** Incidence of AKI by annual eGFR decline between week 4 and LVOT. Data are presented by patients with (Yes) or without (No) an annual eGFR decline of >3 ml/min/1.73 $m^2$ or >5 ml/min/1.73 $m^2$, and by the number (%) and rate per 100 pt-yrs of patients with AKI.
eGFR assessed by MDRD formula. AKI is considered between start of first study drug intake and study drug stop plus 7 days, so could appear before week 4 (while first serum creatinine measurement was planned to be measured at week 4). AKI, acute kidney injury; eGFR, estimated glomerular filtration rate; LVOT, last value on treatment; MDRD, Modification of Diet in Renal Disease; pt-yrs, patient-years.

| **Annual eGFR decline >3 ml/min/1.73 $m^2$** | | | | | | | |
|---|---|---|---|---|---|---|---|
| **No** | | | | **Yes** | | | |
| **Placebo (N=1785)** | | **Empagliflozin (N=3863)** | | **Placebo (N=188)** | | **Empagliflozin (N=134)** | |
| n (%) | Rate per 100 pt-yrs | n (%) | Rate per 100 pt-yrs | n (%) | Rate per 100 pt-yrs | n (%) | Rate per 100 pt-yrs |
| 29 (1.6) | 0.64 | 33 (0.9) | 0.32 | 0 | 0 | 1 (0.7) | 0.26 |
| **Annual eGFR decline >5 ml/min/1.73 $m^2$** | | | | | | | |
| **No** | | | | **Yes** | | | |
| **Placebo (N=1929)** | | **Empagliflozin (N=3954)** | | **Placebo (N=44)** | | **Empagliflozin (N=43)** | |
| n (%) | Rate per 100 pt-yrs | n (%) | Rate per 100 pt-yrs | n (%) | Rate per 100 pt-yrs | n (%) | Rate per 100 pt-yrs |
| 29 (1.5) | 0.59 | 33 (0.8) | 0.31 | 0 | 0 | 1 (2.3) | 0.78 |
